# Suture of Nerves

**Published:** 1868-07

**Authors:** 


Suture of Nerves.—At the Society of Surgery, M. Paulet has read a very elaborate paper on the Immediate and Indirect Consequences of Traumatic Lesions of Nerves. Two cases here recently have excited interest in this subject. In one, M. Langier united by suture the cut ends of the median nerve; and on the same day there was a commencement of restored sensibility and voluntary movement. In the other, M. Richet demonstrated, notwithstanding the complete section of the median nerve, that tactile sensibility persisted in the thumb, index, medium, and ring fingers. These clinically observed facts are entirely opposed to the teachings of experimental physiologists who have divided and resected nerves. To explain them, reference has been made to peripheral anastomoses, such as M. Robin has pointed out between the median and radial, for the nervous filaments distributed to the tactile corpuscles. But M. Paulet points out that, if the explanation lies here, the function should have been carried on by them immediately after the section of the nerves, not slowly re-established. After a very long literary research and a great number of experiments, he confesses himself unable to elucidate the difficulty and discord.—*British Medical Journal,* May 16, 1868.

